# The effect of different anaesthetics on echocardiographic evaluation of diastolic dysfunction in a heart failure with preserved ejection fraction model

**DOI:** 10.1038/s41598-020-72924-5

**Published:** 2020-09-24

**Authors:** Ilona Cuijpers, Paolo Carai, Pedro Mendes-Ferreira, Steven J. Simmonds, Paul Mulder, Daniela Miranda-Silva, Daria De Giorgio, Peter Pokreisz, Stephane Heymans, Elizabeth A. V. Jones

**Affiliations:** 1 Centre for Molecular and Vascular Biology, Department of Cardiovascular Sciences, KU Leuven, Herestraat 49, Bus 911, 3000 Leuven, Belgium; 2 Department of Cardiology, CARIM School for Cardiovascular Diseases, Maastricht University, Universiteitssingel 50, 6229 ER Maastricht, The Netherlands; 3 Department of Chronic Diseases, Metabolism and Aging, Respiratory Division, KU Leuven, Herestraat 49, Bus 707, 3000 Leuven, Belgium; 4 Department of Physiology and Cardiothoracic Surgery, Cardiovascular R&D Unit, Faculty of Medicine, University of Porto, Alameda Prof. Hernâni Monteiro, 4200-319 Porto, Portugal; 5 Faculty of Medicine and Pharmacy, UMR1096, Institut National de La Santé Et de La Recherche Médicale (Inserm), 22 Boulevard Gambetta, 76183 Rouen, France; 6 Department of Cardiovascular Medicine, Laboratory of Cardiopulmonary Pathophysiology, Istituto Di Ricerche Farmacologiche “Mario Negri” IRCCS, Via Mario Negri 2, 201566 Milan, Italy; 7 ICIN-Netherlands Heart Institute, Holland Heart House, Moreelsepark 1, 3511 EP Utrecht, The Netherlands

**Keywords:** Heart failure, Ultrasound, Experimental models of disease, Preclinical research

## Abstract

Heart failure with preserved ejection fraction (HFpEF) is currently untreated. Therapeutics development demands effective diagnosis of diastolic dysfunction in animal models mimicking human pathology, which requires appropriate anaesthetics. Here, we investigated which anaesthetic, ketamine/xylazine or isoflurane, could be used to reveal diastolic dysfunction in HFpEF-diseased obese ZSF1 rats by echocardiography. First, diastolic dysfunction was confirmed by pressure-volume loops in obese compared to lean control ZSF1 rats. In echocardiography, ketamine/xylazine, unlike isoflurane, was able to demonstrate impaired relaxation in obese ZSF1 rats, as reflected by impaired early (E) and late (A) filling peak velocities, decreased E/A ratio, and a prolonged deceleration and isovolumic relaxation time. Interestingly, ketamine/xylazine induced a wider separation of both tissue and pulsed wave Doppler-derived echocardiographic waves required for diastolic dysfunction diagnosis, potentially by reducing the heart rate (HR), while isoflurane resulted in merged waves. To assess whether HR-lowering alone explained the differences between the anaesthetics, echocardiography measurements under isoflurane with and without the HR-lowering drug ivabradine were compared. However, diastolic dysfunction could not be diagnosed in ivabradine-treated obese ZSF1 rats. In summary, ketamine/xylazine compared to isoflurane is the anaesthetic of choice to detect diastolic dysfunction by echocardiography in rodent HFpEF, which was only partly mediated by HR-lowering.

## Introduction

Heart failure with preserved ejection fraction (HFpEF) is a complex heterogeneous cardiovascular syndrome characterised by diastolic dysfunction and cardiac remodelling (stiffening, inflammation, and hypertrophy) in the presence of a preserved ejection fraction (≥ 50%). Due to the ageing population, as well as an improved survival of patients with comorbidities, such as obesity, type 2 diabetes mellitus (T2DM), and hypertension, the prevalence of HFpEF is steadily rising, accounting for more than 50% of incident HF overall^1^. Despite the improved management of heart failure with reduced ejection fraction (HFrEF) over the last two decades, little advancement has been made in identifying evidence-based treatments for HFpEF. Treatment of HFpEF has been especially complicated by incomplete understanding of the pathophysiology, patient population heterogeneity, and inadequate diagnosis^2^.

Animal models are essential for understanding HFpEF disease progression and treatment. Obese Zucker fatty spontaneously hypertensive heart failure F1 hybrid (ZSF1) rats are an established HFpEF model associated with cardiometabolic risks, including obesity, T2DM, and hypertension^3^. Echocardiography is crucial for the differential diagnosis of diastolic function in these rats, however the use of anaesthetics is required for reduction of stress and reliable and reproducible recordings^4^. Anaesthetics are well known to affect cardiac preload, afterload, myocardial contractility, and hemodynamics (e.g. heart rate)^5^. However, their effects on cardiac function, including systolic function, hemodynamics, left ventricular dimensions, and especially diastolic function, have never been investigated in a HFpEF rodent model. Accordingly, this study examined the effect of the most commonly used anaesthetics for rodent models, isoflurane and ketamine/xylazine, on cardiac function during echocardiography acquisition in obese and lean control ZSF1 rats.

## Results

### Diastolic dysfunction in obese ZSF1 rats confirmed by pressure-volume loops

**Figure 1:**
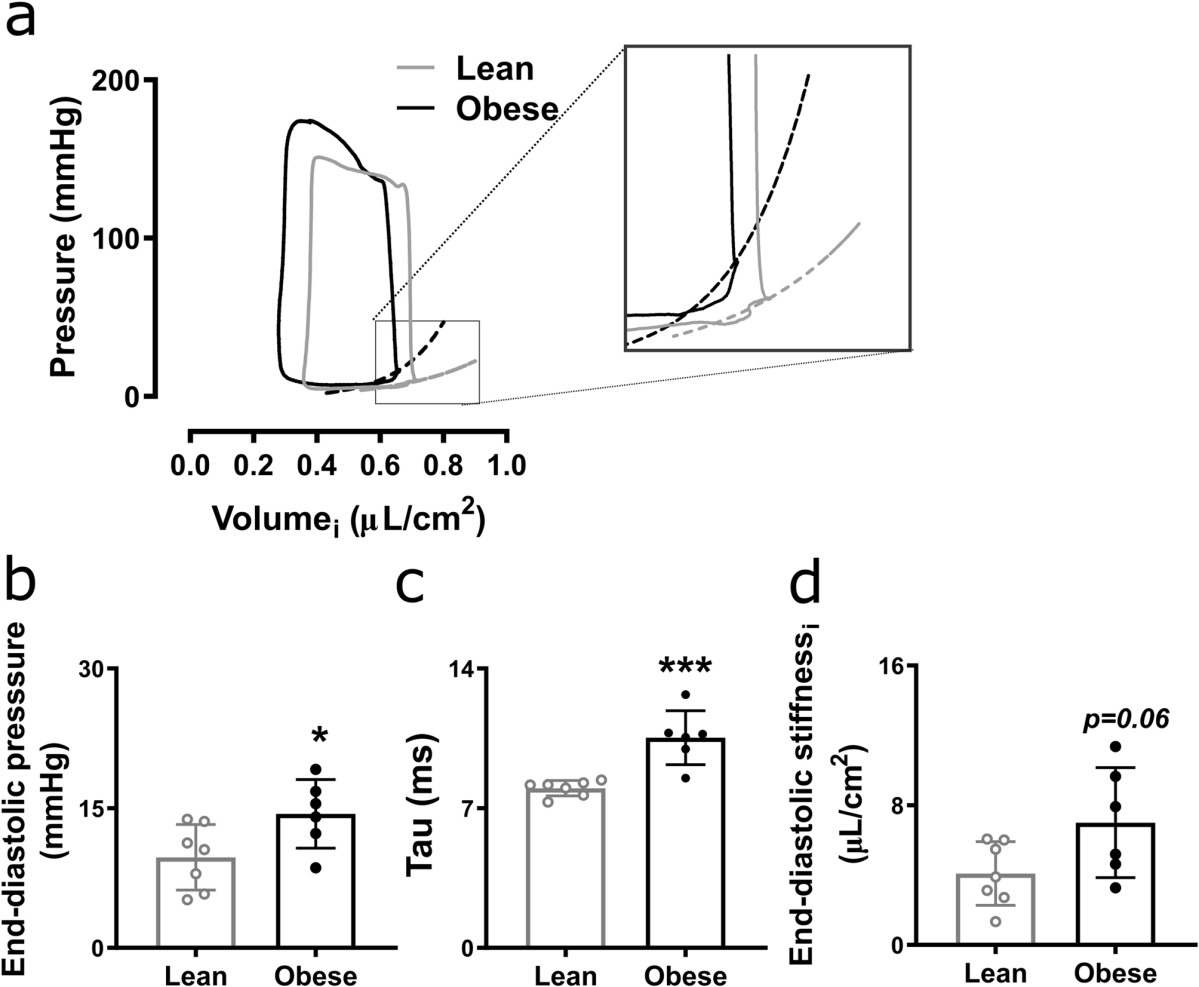
Diastolic dysfunction in obese ZSF1 rats assessed by pressure-volume loops. Pressure-volume loops indexed for body surface area (**a**), end-diastolic pressure (**b**), tau (**c**), and end-diastolic stiffness indexed for body surface area (**d**) in 6 obese and 7 lean ZSF1 rats at 21 weeks. Values are presented as mean ± SD. *P < 0.05 and *** < 0.001. i, indexed for body surface area, calculated as 9.1 * body weight in grams^2/3^.

To confirm that obese ZSF1 rats developed diastolic dysfunction, we performed golden standard pressure-volume loops in obese and lean control ZSF1 rats at 21 weeks. Obese ZSF1 rats showed a significantly decreased heart rate and increased non-body surface area (BSA)-indexed stroke volume compared to lean ZSF1 rats, while BSA-indexed stroke volume and cardiac output and index were similar between lean and obese ZSF1 rats (Table 1). Furthermore, obese ZSF1 rats showed a worsened hypertensive profile, reflected by significantly increased systolic and mean arterial pressure, while diastolic arterial pressure was modestly, albeit not significantly (*p* = 0.06), increased compared to lean ZSF1 rats (Table 1). Obese ZSF1 rats had an increased end-diastolic pressure and tau, confirming an impaired diastolic function in obese compared to lean ZSF1 rats (Fig. 1a–c and Table 1). BSA-indexed end-diastolic stiffness (also called end-diastolic elastance) was modestly, although non-significantly (*p* = 0.06), increased in obese compared to lean ZSF1 rats (Fig. 1d and Table 1). Similarly, non-indexed end-diastolic stiffness was not significantly different between lean and obese ZSF1 rats (Table 1). Peak of pressure decline and BSA-indexed and non-indexed end-diastolic volumes were similar in lean and obese ZSF1 rats (Table 1). Systolic parameters, including ejection fraction (> 50%), peak rate of pressure rise, preload recruitable stroke work, and BSA-indexed and non-indexed end-systolic volume, arterial elastance, and end-systolic stiffness (also called end-systolic elastance) were comparable in lean and obese ZSF1 rats (Table 1). In summary, pressure-volume loops confirmed diastolic dysfunction in presence of a preserved ejection fraction in 21-week-old obese ZSF1 rats compared to age-matched lean ZSF1 rats.

**Table 1:** Pressure-volume loops in left ventricle and aorta of lean and obese ZSF1 rats at 21 weeks.

Parameter	Lean (n = 7)	Obese (n = 6)	*P* value
**Baseline**			
Body surface area (cm^2^)	470 ± 33	601 ± 15	**0.0006**
Heart rate (bpm)	313 ± 18	277 ± 33	**0.0307**
Stroke volume (µl)	178 ± 11	221 ± 13	**0.0271**
Stroke volume, _i_ (µl/cm^2^)	0.38 ± 0.07	0.37 ± 0.02	0.7207
Cardiac output (ml/min)	56 ± 7.1	61 ± 12	0.3167
Cardiac index (ml/min/cm^2^)	0.12 ± 0.02	0.10 ± 0.02	0.1623
Ejection fraction (%)	56 ± 7.8	62 ± 14	0.2921
End-systolic pressure (mmHg)	143 ± 13	169 ± 17	**0.0098**
End-systolic volume (µl)	174 ± 47	187 ± 83	0.7265
End-systolic volume, _i_ (µl/cm^2^)	0.37 ± 0.10	0.31 ± 0.14	0.3884
Peak of pressure rise (mmHg/s)	8532 ± 1127	8695 ± 556	0.7545
Arterial elastance (mmHg/µl)	0.82 ± 0.19	0.75 ± 0.13	0.4316
Arterial elastance, _i_ (mmHg/µl/cm^2^)	389 ± 96	449 ± 80	0.2495
End-diastolic pressure (mmHg)	9.7 ± 3.5	14.4 ± 3.68	**0.0393**
End-diastolic volume (µl)	335 ± 21	385 ± 30	0.1985
End-diastolic volume, _i_ (µl/cm^2^)	0.72 ± 0.14	0.64 ± 0.12	0.3272
Peak of pressure decline (mmHg/s)	− 9758 ± 1382	− 9231 ± 1003	0.4557
Tau Logistic (ms)	8.0 ± 0.4	11 ± 1.4	**0.0006**
**Occlusion**			
Preload recruitable stroke work (mmHg)	75 ± 24	75 ± 36	0.9992
End-systolic stiffness (mmHg/µl)	0.62 ± 0.22	0.50 ± 0.23	0.3896
End-systolic stiffness, _i_ (mmHg/µl/cm^2^)	289 ± 107	301 ± 137	0.8696
End-diastolic stiffness (mmHg/µl)	0.009 ± 0.004	0.01 ± 0.0005	0.2763
End-diastolic stiffness, _i_ (µl/cm^2^)	4.1 ± 1.8	7.0 ± 3.2	0.0616
**Aortic parameters**			
Systolic arterial pressure (mmHg)	143 ± 13	167 ± 16	**0.0131**
Diastolic arterial pressure (mmHg)	99 ± 15	116 ± 15	0.0632
Mean arterial pressure (mmHg)	121 ± 15	141 ± 15	**0.0339**

Values are presented as mean ± SD. _i_, indexed for body surface area, calculated as 9.1 * body weight in grams^2/3^.

### Diastolic dysfunction in obese ZSF1 rats is detectable by echocardiography using ketamine/xylazine

**Figure 2:**
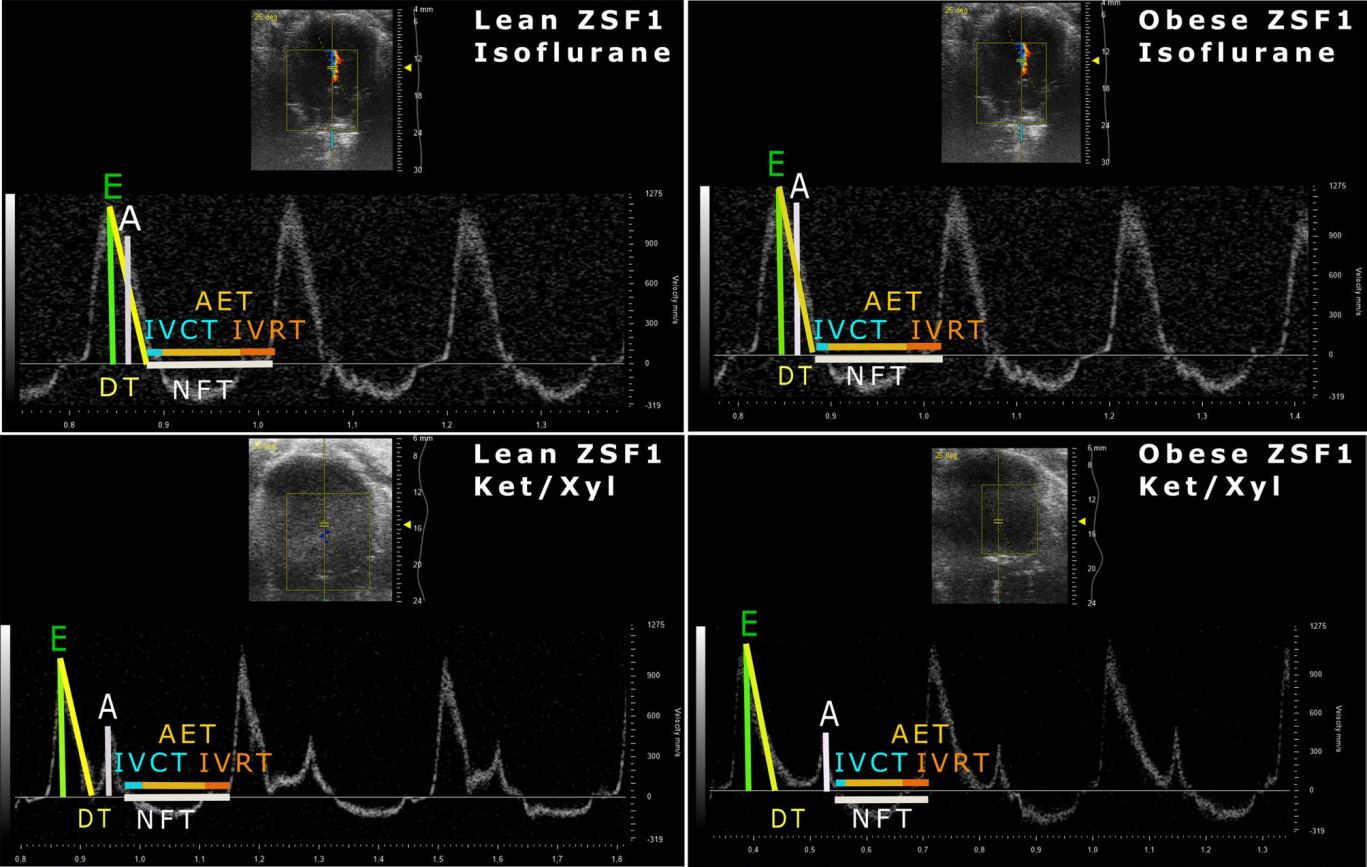
Ketamine/xylazine resulted in a more distinct separation of pulsed wave Doppler waves in both obese and lean ZSF1 rats. Pulsed wave Doppler images of isoflurane- and ketamine/xylazine-anaesthetised 20-week-old lean and obese ZSF1 rats (n = 7 per group). A, late mitral inflow peak velocity; AET, aortic ejection time; DT, mitral valve deceleration time; E, early mitral inflow peak velocity; IVCT, isovolumic contraction time; IVRT, isovolumic relaxation time; Ket/Xyl, ketamine/xylazine; NFT, non-flow time.

**Figure 3:**
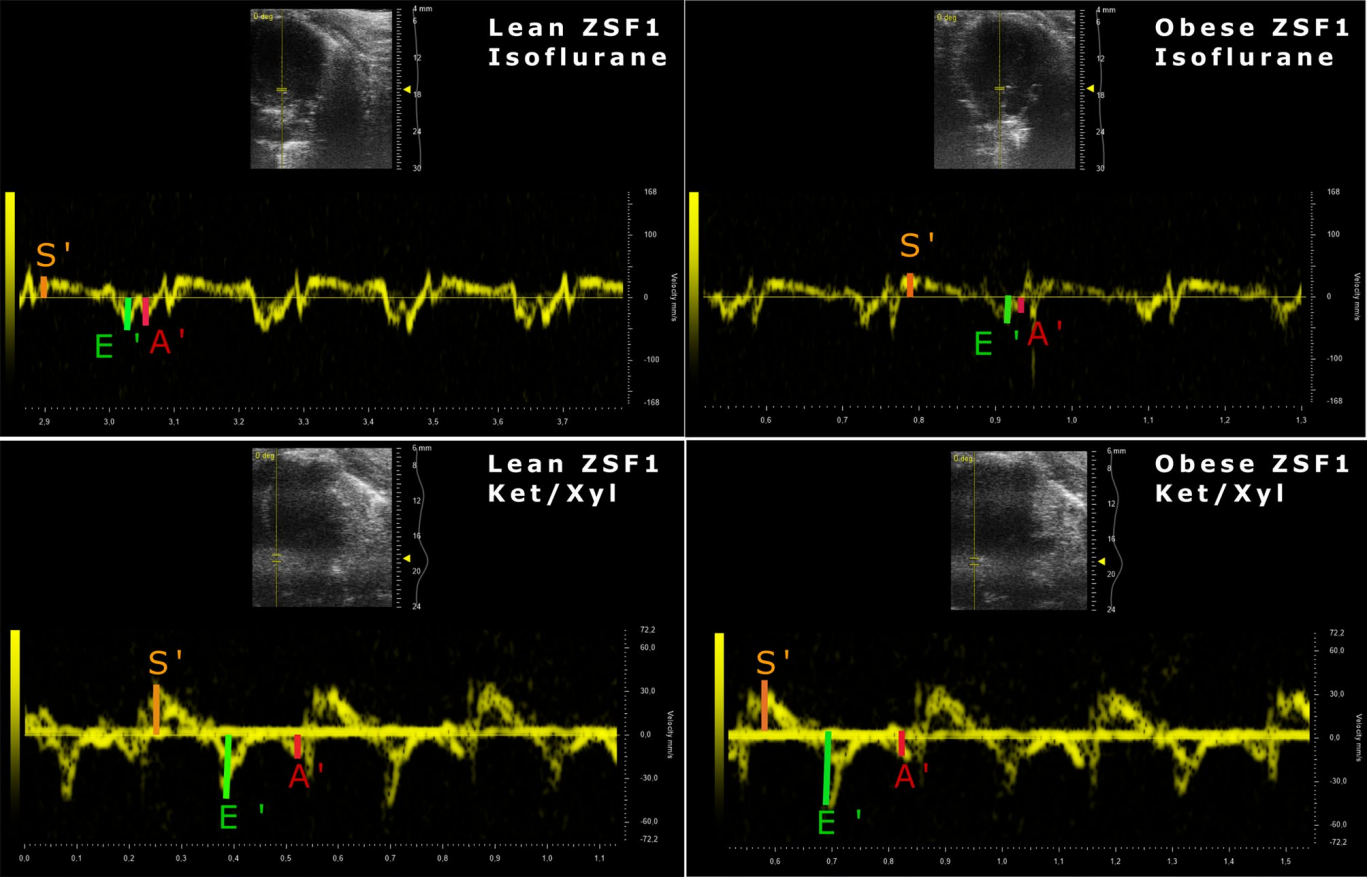
Ketamine/xylazine induced a more distinct separation of tissue Doppler waves in lean and obese ZSF1 rats. Tissue Doppler images of 20-week-old isoflurane- and ketamine/xylazine-anaesthetised lean and obese ZSF1 rats (n = 7 per group). A′, late mitral annulus peak velocity; E′, early diastolic mitral annulus peak velocity; Ket/Xyl, ketamine/xylazine; S′, systolic peak velocity.

**Figure 4:**
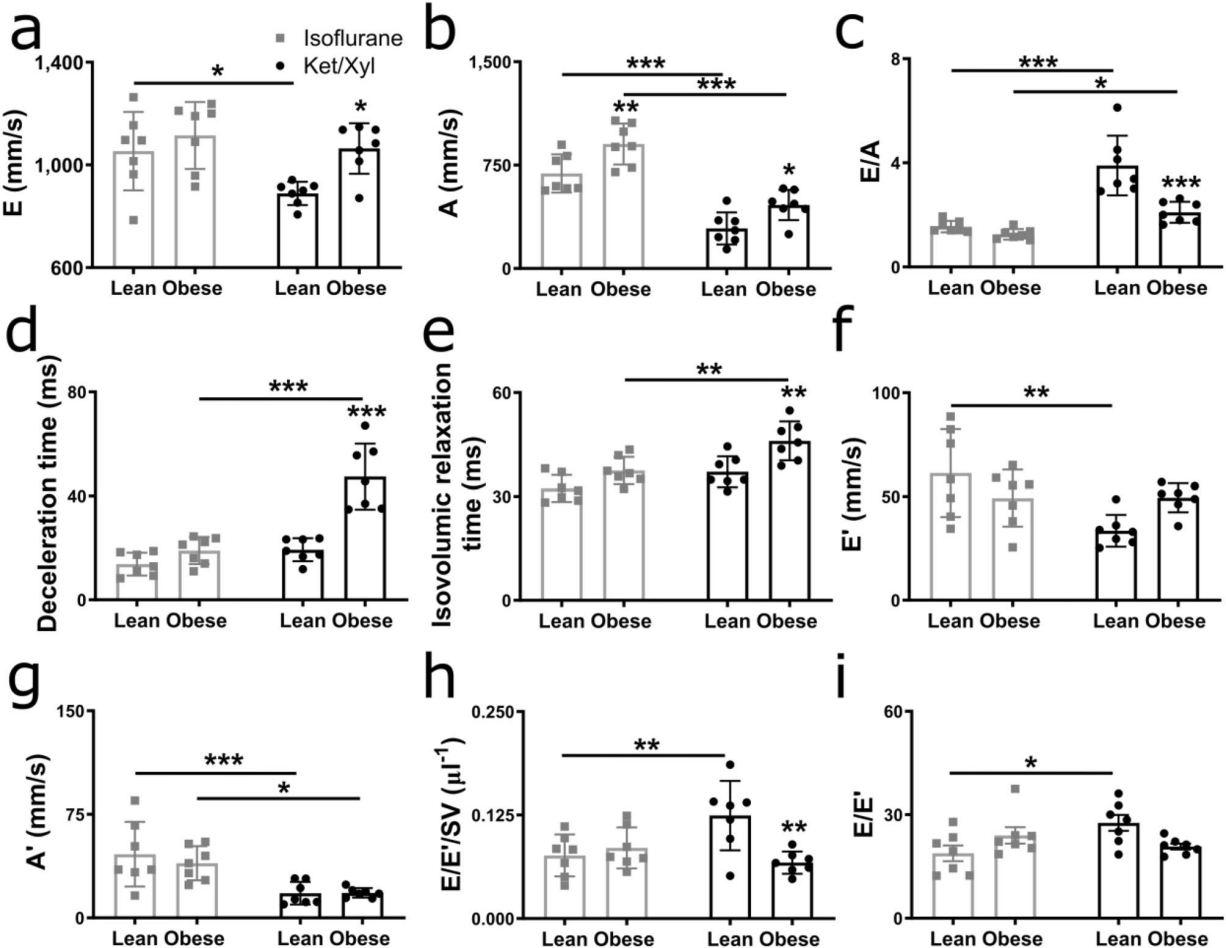
Diastolic dysfunction in obese ZSF1 rats is detectable when ketamine/xylazine is used as an anaesthetic. E and A peak velocities (**a**, **b**), E/A ratio (**c**), mitral valve deceleration time (**d**), isovolumic relaxation time (**e**), E′ and A′ peak velocities (**f**, **g**), and E/E′/SV and E/E′ ratios (**h**, **i**) in 20-week-old isoflurane- or ketamine/xylazine-anaesthetised lean and obese ZSF1 rats (n = 7 per group). Values are presented as mean ± SD. *P < 0.05, ** < 0.01, and *** < 0.001. A, late mitral inflow peak velocity; A′, late mitral annulus peak velocity; E, early mitral inflow peak velocity; E′, early diastolic mitral annulus peak velocity; Ket/Xyl, ketamine/xylazine; SV, stroke volume.

To assess whether diastolic dysfunction could be diagnosed by echocardiography using isoflurane or ketamine/xylazine, we performed pulsed wave and tissue Doppler echocardiography in lean and obese ZSF1 rats at 20 weeks. A clear separation of both early (E) and late (A) mitral inflow peak velocity waves in pulsed wave Doppler and early (E′) and late (A′) diastolic mitral annulus peak velocity waves in tissue Doppler was observed in both lean and obese ZSF1 rats anaesthetised with ketamine/xylazine (Figs. 2 and 3, lower panels). In contrast to ketamine/xylazine, anaesthesia with isoflurane superimposed E and A waves, as well as E′ and A′ waves, on each other, thereby impeding the reliable assessment of diastolic function (Figs. 2 and 3, upper panels). Ketamine/xylazine significantly decreased E peak velocities in lean ZSF1 rats, while decreasing A peak velocities in both lean and obese ZSF1 rats, resulting in significantly increased E/A ratios in both lean and obese ZSF1 rats compared to isoflurane-anaesthetised ZSF1 rats (Fig. 4a–c). In obese ZSF1 rats, ketamine/xylazine increased mitral valve deceleration time, isovolumic relaxation time, and non-flow time compared to isoflurane (Fig. 4d,e and Supplementary Table S1). Furthermore, ketamine/xylazine decreased E′ peak velocities in lean ZSF1 rats, while it reduced A′ peak velocities in both lean and obese ZSF1 rats compared to isoflurane (Fig. 4f,g). In obese ZSF1 rats, ketamine/xylazine increased E′/A′ compared to isoflurane, while in lean ZSF1 rats, it increased both the operant diastolic elastance (E/E′/SV) and the E/E′ ratio (Fig. 4h,i and Supplementary Table S1). Importantly, diastolic dysfunction could only be diagnosed when using ketamine/xylazine in obese ZSF1 rats, as reflected by a decreased E/A ratio, operant diastolic elastance (E/E′/SV), and myocardial performance index, and increased E and A peak velocities, mitral valve deceleration time, isovolumic relaxation time, non-flow time, and E′/A′ ratio compared to lean ZSF1 rats (Fig. 4a–e,h and Supplementary Table S1). In contrast to ketamine/xylazine, isoflurane only increased A peak velocities in obese compared to lean ZSF1 rats, showing the inability to detect diastolic dysfunction when using isoflurane as an anaesthetic (Fig. 4a–i and Supplementary Table S1). Taken together, diastolic dysfunction in obese ZSF1 rats is detectable when using ketamine/xylazine but cannot be identified when isoflurane is administered as an anaesthetic.

### Ketamine/xylazine has a cardiodepressive effect without affecting systolic parameters in both obese and lean ZSF1 rats

**Figure 5:**
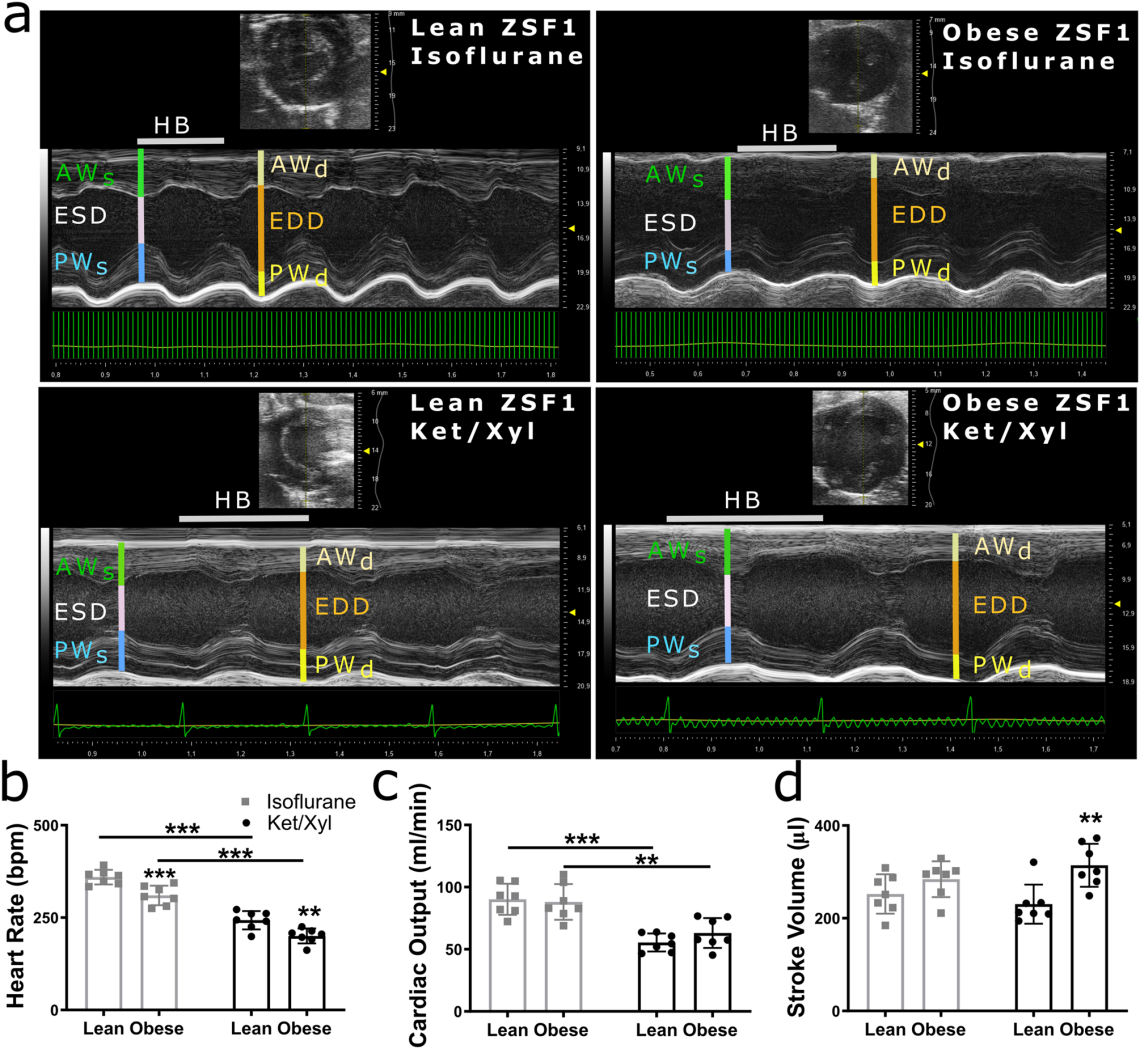
Ketamine/xylazine was cardiodepressive in both lean and obese ZSF1 rats. M-mode images of 20-weeks-old isoflurane- and ketamine/xylazine-anaesthetised lean and obese ZSF1 rats (n = 7 per group) (**a**). Heart rate (**b**), cardiac output (**c**), and stroke volume (**d**) in 20-week-old isoflurane- or ketamine/xylazine-anaesthetised lean and obese ZSF1 rats. Values are presented as mean ± SD. **P < 0.01 and *** < 0.001. AW, anterior wall; EDD, end-diastolic diameter; ESD, end-systolic diameter; HB, heartbeat; Ket/Xyl, ketamine/xylazine; PW, posterior wall.

We subsequently investigated the anaesthetic’s effects on left ventricular dimensions, hemodynamics, and systolic function. Ketamine/xylazine had cardiodepressive effects in both lean and obese ZSF1 rats, as reflected by a drop in heart rate and cardiac output (Fig. 5a–c). Similar to pressure-volume loops, a decreased heart rate was observed in obese compared to lean ZSF1 rats, regardless of the type of anaesthetic (Fig. 5b). Stroke volume was only increased in ketamine/xylazine-anaesthetised obese compared to lean ZSF1 rats, while correction for BSA resulted in similar stroke volumes in all groups (Fig. 5d and Supplementary Table S1). Cardiac index was significantly reduced in isoflurane-anaesthetised obese compared to lean ZSF1 rats, while ketamine/xylazine significantly reduced cardiac index in both lean and obese ZSF1 rats compared to isoflurane (Supplementary Table S1).

Parameters of systolic function (ejection fraction, fractional shortening, systolic peak wave, and isovolumic contraction time) and anterior wall thickness in systole and end-systolic diameter were not affected by the type of anaesthetic (Supplementary Table S1 and Fig. S1a–d). Ketamine/xylazine significantly increased measurements of posterior wall thickness in systole and end-diastolic diameter in obese compared to lean ZSF1 rats, while lean ZSF1 rats showed an increased anterior wall thickness in diastole when measured using ketamine/xylazine compared to isoflurane (Supplementary Table S1 and Fig. S1e–g). Measurements of posterior wall thickness in diastole and non-indexed and indexed end-systolic and -diastolic volumes were not affected by the type of anaesthetic (Supplementary Table S1 and Fig. S1h–j). These findings demonstrated that ketamine/xylazine had a cardiodepressive effect but did not differently affect systolic function and measurements of left ventricular volumes.

### Heart rate-lowering alone does not allow diagnosis of diastolic dysfunction in isoflurane-anaesthetised obese ZSF1 rats

**Figure 6:**
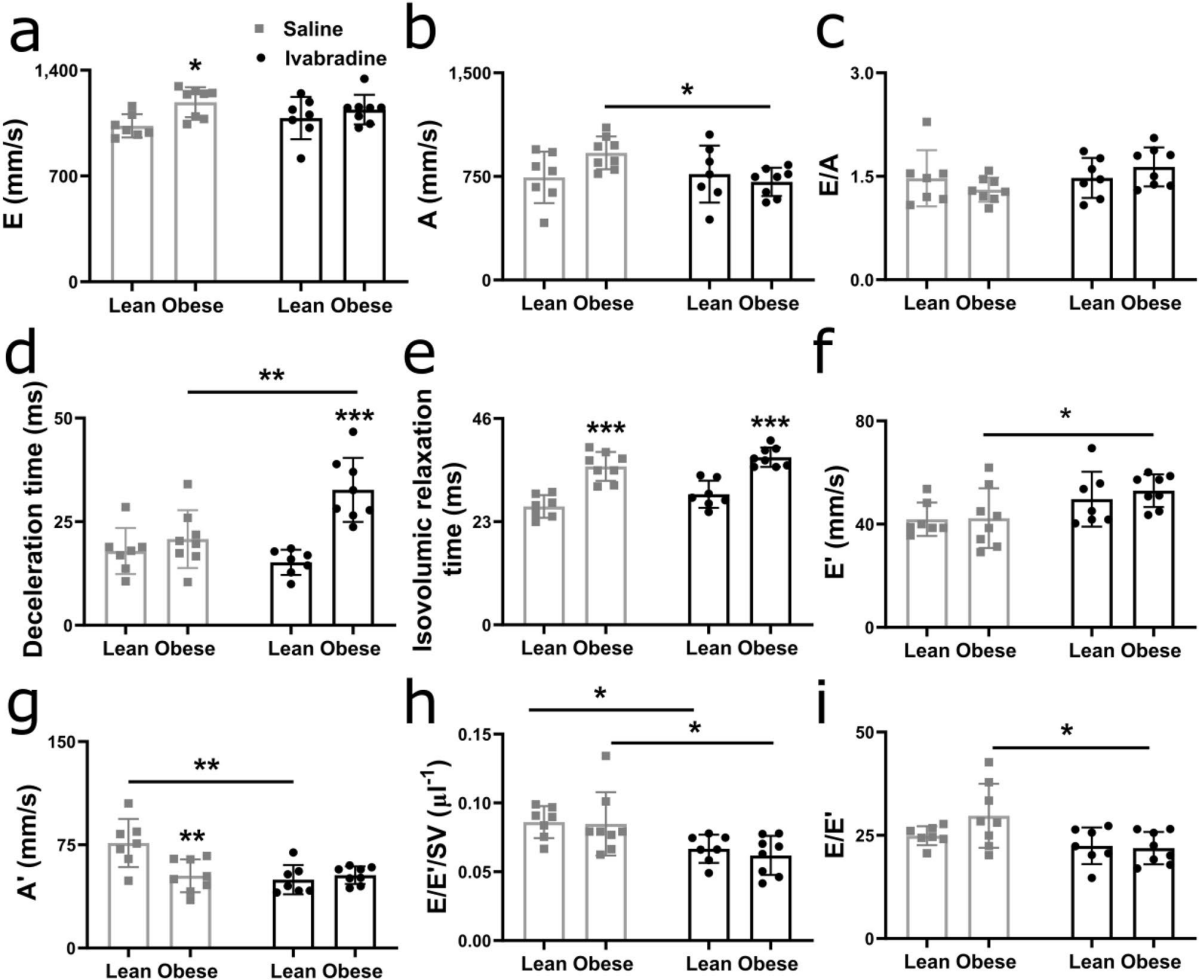
Heart rate reduction does not allow diagnosis of diastolic dysfunction in obese ZSF1 rats. E and A peak velocities (**a**, **b**), E/A ratio (**c**), mitral valve deceleration time (**d**), isovolumic relaxation time (**e**), E′ and A′ peak velocities (**f**, **g**), and E/E′/SV and E/E′ ratios (**h**, **i**) in 20-week-old isoflurane-anaesthetised lean and obese ZSF1 rats (n = 7 and 8 per group, respectively) administered with ivabradine or saline (vehicle control). Values are presented as mean ± SD. *P < 0.05, ** < 0.01, and *** < 0.001. A, late mitral inflow peak velocity; A′, late mitral annulus peak velocity; E, early mitral inflow peak velocity; E′, early diastolic mitral annulus peak velocity; Ket/Xyl, ketamine/xylazine; SV, stroke volume.

To assess whether the negative chronotropic (heart rate-lowering) effects of ketamine/xylazine were responsible for the ability to detect diastolic dysfunction in obese ZSF1 rats, we used ivabradine – having solely negative chronotropic effects – along with isoflurane anaesthesia. Ivabradine significantly reduced the heart rate in both lean and obese ZSF1 rats, while in obese ZSF1 rats, it reduced cardiac output compared to saline, all showing its heart rate-reducing effect (Supplementary Table S2). Ivabradine decreased ejection fraction and fractional shortening due to an increased end-diastolic volume in obese compared to lean ZSF1 rats, but this decrease in ejection fraction was still within the definition of “preserved ejection fraction” (≥ 50%; Supplementary Table S2). Furthermore, other systolic parameters, including isovolumic contraction time and systolic peak wave velocity, were similar in ivabradine-treated lean and obese ZSF1 rats (Supplementary Table S2). Interestingly, separation of pulsed wave and tissue Doppler waves was improved in both lean and obese ZSF1 rats treated with ivabradine. However, E, A, and E/A ratio were similar between ivabradine-treated obese and lean ZSF1 rats, indicating the inability to detect diastolic dysfunction (Fig. 6a–c). Ivabradine-treated obese ZSF1 rats showed an increased mitral valve deceleration time, isovolumic relaxation time, non-flow time, and E′/A′ ratio and a reduced myocardial performance index compared to lean ZSF1 rats (Fig. 6d,e and Supplementary Table S2). Furthermore, E′, A′, E/E′/SV, and E/E′ were similar in lean and obese ZSF1 rats treated with ivabradine (Fig. 6f–i). Thus, despite improved separation of the waves in pulsed wave and tissue Doppler, heart rate reduction did not result in the detection of diastolic dysfunction in isoflurane-anaesthetised obese ZSF1 rats.

## Discussion

Animal models allow us to understand cardiac molecular, cellular, physiological, and functional changes during HFpEF development, leading to the discovery of novel therapeutics. Echocardiography is widely used for the diagnosis of pre-clinal and clinical HFpEF with the advantage of being non-invasive, safe, repeatable, widely available, and inexpensive. Anaesthetics usage during echocardiography acquisition in rodent models and clinical perioperative phases is generally accepted, despite their poorly understood mechanism and impact on cardiac function. While all anaesthetics have some hemodynamic effects, the key is to identify the anaesthetic that can be used to diagnose the disease of interest. The best clinical practice to perform echocardiography in humans is to preserve the heart rate in a normal range between 60–100 bpm for adults. In rats, the normal heart rate is on average around 300–450 bpm, consequently the best strategy in rats is not necessarily the same as in humans. In fact, our results show that ketamine/xylazine improved the ability to diagnose diastolic dysfunction in obese ZSF1 rats over isoflurane. The inability to detect diastolic dysfunction in obese ZSF1 rats, when using isoflurane in combination with the heart rate-lowering drug ivabradine, showed that the detection of diastolic dysfunction in obese ZSF1 rats using ketamine/xylazine is not due to a pure chronotropic effect, but is also mediated by ketamine/xylazine’s inotropic effects.

Ketamine/xylazine allowed for a clear separation of both E and A waves in pulsed wave Doppler and E′ and A′ waves in tissue Doppler compared to isoflurane. Importantly, the consequence of inaccurate separation of these waves, includes possible overestimation of A and A′ peak velocities, while underestimating mitral valve deceleration time and E/A ratio. This inaccurate separation translated in the inability to reliably diagnose diastolic dysfunction in isoflurane-anaesthetised obese ZSF1 rats. We showed that ketamine/xylazine, unlike isoflurane, was able to demonstrate diastolic dysfunction in obese ZSF1 rats. Ketamine/xylazine significantly decreased A peak velocities in both lean and obese ZSF1 rats and E peak velocities in lean ZSF1 rats, resulting in increased E/A ratios in lean and obese ZSF1 rats compared to isoflurane-anaesthetised rats. In rats with chronic aortic valve regurgitation, ketamine/xylazine (0.1 and 0.75 mg/kg, respectively) significantly decreased E peak velocities, while modestly, albeit non-significantly, decreasing A peak velocities (*p* = 0.05) and E/A ratio (*p* = 0.07) compared to 1.5% isoflurane (Supplementary Table S3)^6^. In healthy Fischer 344 rats, ketamine/xylazine increased isovolumic relaxation time without affecting E and A peak velocities, E/A ratio, and mitral valve deceleration time compared to 1.5% isoflurane (Supplementary Table S3)^7^. In Wistar rats, ketamine/xylazine (0.1 and 0.75 mg/kg, respectively) reduced E and A peak velocities without affecting E/A ratio compared to 1.5% isoflurane, while a higher dose of ketamine/xylazine (40 and 8 mg/kg, respectively) decreased A and preserved E, resulting in an increased E/A ratio compared to rats anaesthetised with 1.5% isoflurane (Supplementary Table S3)^6,8^. In preoperative echocardiography, inhaled anaesthetics (sevoflurane, isoflurane, and desflurane) improved diastolic function by decreasing E and A peak velocities in patients with diastolic dysfunction^9–11^, all providing a possible explanation for the inability to detect diastolic dysfunction when using isoflurane.

Heart rate can affect diastolic function and this is one of the reasons that waking heart rates are preferred for echocardiography measurements. In our study, ketamine/xylazine had cardiodepressive effects (reduced heart rate and cardiac output), but did not affect ejection fraction, as shown before (Supplementary Table S3)^6–8,12,13^. Therefore, the improved diagnosis using ketamine/xylazine could be the result of a decreased heart rate (negative chronotropy) and/or cardiac contraction (negative inotropy). However, while the selective negative chronotropic drug ivabradine induced an improved separation of pulsed wave and tissue Doppler waves, diastolic dysfunction could not be detected in isoflurane-anaesthetised obese ZSF1 rats. This indicates that the detection of diastolic dysfunction in obese ZSF1 rats using ketamine/xylazine is not due to a pure chronotropic effect, but that both negative chronotropic and inotropic effects of ketamine/xylazine are required to unveil diastolic dysfunction. This inotropic effect could be mediated by the interference of anaesthetics with the intracellular calcium flow and re-uptake in the sarcoplasmic reticulum (SR). Xylazine acts as an alpha 2 receptor agonist, reducing cardiac intracellular cyclic adenosine monophosphate (cAMP), the influx of extracellular calcium, the SR-mediated uptake of calcium, and the sensitivity of the contractile proteins to calcium, ultimately leading to reduced cardiac contractility^14^. Ketamine has both a positive inotropic effect, by increasing myocardial calcium influx, and negative inotropic effect by impairing calcium flux in the SR^15,16^. In addition, ketamine affects a wide range of other processes, such as non-competitive blockage of N-methyl-D-aspartate (NMDA) channels and nicotinic receptors, activation of L-arginine/nitric oxide (NO)/cyclic guanosine-mono-phosphate (cGMP) signalling, and promotion of the release of noradrenaline^17–19^, all indicating that ketamine/xylazine can affect diastolic function by a wide range of effects.

Overall, given all our data, depth of anaesthesia is critical for accurate assessment of diastolic dysfunction. The doses proposed here should not be blindly applied to other strains, sexes, or ages. In other rodent models, a dose–response curve needs to be performed, with special attention to obese models, as anaesthetics can accumulate in adipose tissue layers. Anaesthetic induction and image acquisition should always be performed within the same range of time for all the experimental animals. Electrocardiogram (ECG) recording can be used to monitor the breathing rate, which is a measurement for anaesthetic depth; a reduced breathing rate and superficial breathing indicate a too light anaesthesia depth, while an increased breathing rate and gasping (heavily breathing) indicate a too deep anaesthesia depth. In addition, ECG acquisition is crucial for ensuring the reduction in heart rate, which is required for a proper separation of the pulsed wave and tissue Doppler waves. The heart rate should be monitored during the whole echocardiography procedure. A heart rate above 300 bpm does not allow the separation of the waves in pulsed wave and tissue Doppler. Optimally the heart rate should be between 220–260 bpm to allow the separation of the waves in pulsed wave and tissue Doppler.

We have presented both indexed and non-indexed volumes, as it is debated whether indexing is appropriate for the diagnosis of diastolic dysfunction in rodents. BSA is the most widely used parameter for indexing cardiac volumes in preclinical and clinical settings^20,21^, however, it has several limitations. First of all, correct BSA calculation is complex and different formulae are available^22^. Clinical calculations of BSA take into account both height and body mass^22^, however, common rodent calculations are much simpler and only use the rodent’s mass to estimate BSA. Given that the presence of obesity can stunt growth, the simplified calculation of BSA based on purely body mass may underestimate the prevalence of cardiac pathology (e.g. hypertrophy, dilation, etc.) in overweight and obese rodents^20^. Furthermore, animal research is more controlled (e.g. all same age, background, gender etc.) compared to clinical research. As such, indexing for BSA in patients is required to obtain more standardized values, which can be compared between the different experimental groups, but the same concerns may not apply to rodent models.

### Limitations

Different animals were used for each type of anaesthetic, due to concerns that the physiological stress induced by the anaesthetics could affect the measurement of cardiac function, specifically diastolic function. The administration of a single anaesthetic was already difficult, as the obese ZSF1 rats were diseased and susceptible to death. As such, we did not use the same animals for both anaesthetics, even though common practice is to use the same animals. We assessed the effect of isoflurane and ketamine/xylazine only in one HFpEF rodent model, as this is currently the most accepted HFpEF rodent model, which mimics human pathology. We only used male ZSF1 rats, as the model was established in males and few studies have investigated the development in female rats^23^. However, we do not rule out that females might respond differently. Pressure-volume loops were obtained only under isoflurane, as a stable anaesthesia depth is required over a long period of time, and ketamine/xylazine results in an inconstant anaesthesia depth, which gradually declines over time. For echocardiography, only a limited amount of time is required, which ensures that the anaesthesia depth is more stable.

In conclusion, the frequently used anaesthetics isoflurane and ketamine/xylazine differently affect echocardiography-derived cardiac hemodynamics and diastolic function both in HFpEF-diseased obese and control lean ZSF1 rats. Diastolic dysfunction could only be detected in obese ZSF1 rats anaesthetised with ketamine/xylazine. As the prevalence of HFpEF and surgical procedures increase steadily with aging, the impact of anaesthetics on diastolic function should be taken in consideration and further investigated in both animal models and patients.

## Methods

### Experimental design

Experiments were performed according to the European Directive (2010/63/EU) and approved by the Animal Care and Use Committee of KU Leuven (Project 168/2016). 48 male 5-weeks-old ZSF1 rats, including obese and lean control littermates, were obtained from Charles River Laboratories (#strain code 378 and 379, respectively). Obese ZSF1 rats develop metabolic risk induced-HFpEF, characterised by a cluster of comorbidities (obesity, T2DM, and hypertension), increased left ventricular myocardial stiffness and hypertrophy, and a preserved ejection fraction (≥ 50%), thereby mimicking HFpEF in humans^24^. In contrast, lean littermates only develop hypertension, thereby serving as non-HFpEF diseased controls^24^. Animals were housed and acclimated under a 12-h light-dark cycle with access to water and chow diet ad libitum (#V1534-000, ssniff Spezialdiäten GmbH, Germany). At 20 weeks, echocardiography with isoflurane or ketamine/xylazine was performed on lean and obese ZSF1 (n = 7 per group). Fourteen of those animals underwent subsequently pressure-volume loops at 21 weeks. One obese ZSF1 rat died during the pressure-volume loop acquisition. On a different set of animals, including seven lean and eight obese ZSF1 rats per group (kindly provided by Prof. Paul Mulder), echocardiography was performed with or without intraperitoneal (IP) administration of 0.3 mg/kg of the HR-lowering drug ivabradine in combination with 1.5% inhaled isoflurane.

### Pressure-volume loops

Lean and obese ZSF1 rats were anaesthetised with isoflurane, 8% for induction and 2–3.5% for maintenance (Ecuphar, Belgium). Animals were endotracheally intubated (14G) and mechanically ventilated (Rovent, Kent Scientific Corporation, USA) using 100% oxygen, while maintaining positive end-expiratory pressure at 5 cm H_2_O. Proper sedation was confirmed by assessing the pedal withdraw reflex in the hind limbs, and the toe pinch and eyelid reflex^25^.

Body temperature was controlled using a homeothermic blanket system to prevent anaesthesia-induced hypothermia and cardiac electrical activity was monitored using an ECG (Bio Amplifier, FE136, AD Instruments Ltd., UK). Using an open chest approach, a SPR-838 pressure-volume catheter (Millar Instruments, USA) was inserted in the left ventricle through the apex. Fluid loss was compensated by infusion of warmed 0.9% saline at a rate of 32 ml/kg/h through the femoral vein (25G IV catheter, B. Braun Melsungen AG, Germany). Bolus injections of 50 µl hypertonic saline (10% NaCl) were administered to determine parallel conductance. Slope factor α was determined by dividing the pressure-volume loop- and echocardiographic-derived cardiac output. Pressure and volume signals were continuously acquired using a Millar Ultra Pressure Volume system (Millar Instruments, USA), digitally recorded using a Power lab 16/35 data acquisition system, and analysed using Lab Chart 8 (both ADInstruments Ltd., UK). After a 15 min stabilization period, baseline acquisitions were recorded at suspended end-expiration. Transient occlusions of the inferior vena cava using a 3-0 silk lace were achieved to obtain load-independent indexes. Hemodynamic parameters included heart rate, cardiac output, ejection fraction, end-diastolic and -systolic pressures and volumes, logarithmic isovolumic relaxation time constant tau (logistic method), peak rate of pressure rise and decline, preload-recruitable stroke work, arterial elastance, as well as the slope of the end-systolic and -diastolic relationship, called end-systolic and end-diastolic stiffness (elastance). The catheter was subsequently positioned in the ascending aorta to measure systolic and diastolic arterial pressures. Mean arterial pressure was calculated, whereby mean arterial pressure equals 2/3 diastolic arterial pressure plus 1/3 systolic arterial pressure. Heparinized blood was collected for conductance-based volume calibration (#910-1048, Millar Instruments, USA). As the body weight of obese ZSF1 rats is significantly higher than the lean control ZSF1 rats, volumes were indexed for BSA, estimated as 9.1 * body weight in grams^2/3^, to account for differences in body weight between the groups^24,26^. Both the indexed and non-indexed volumes for pressure-volume loops are presented. After the procedure, animals were euthanised by anaesthetics overdose using 50 mg/kg ketamine (Nimatek, Eurovet Animal Health BV, Netherlands) and 5 mg/kg xylazine (Xyl-M, V.M.D. nv/sa, Belgium) dissolved in 0.9% NaCl. Death was confirmed by absence of an ECG signal.

### Echocardiography

Lean and obese ZSF1 rats were randomized for echocardiography and anaesthetised with either an IP injection of 50 mg/kg ketamine (Nimatek, Eurovet Animal Health BV, Netherlands) and 5 mg/kg xylazine (Xyl-M, V.M.D. nv/sa, Belgium) (Ket/Xyl) dissolved in 0.9% NaCl (n = 7/group) or 5% inhaled isoflurane (Ecuphar, Belgium) for induction followed by 1.5% inhaled isoflurane for maintenance (n = 7/group).

To assess the effect of heart rate lowering, 7 lean and 8 obese ZSF1 rats were randomly assigned to isoflurane (5% for induction and 1.5% for maintenance) with IP administration of 0.3 mg/kg ivabradine in 0.9% saline (Procoralan, Servier, France) or 0.9% saline (vehicle control). Echocardiography was performed during two days with a washout period for ivabradine for at least 24 h after the previous acquisition.

Similar to pressure-volume loops, proper sedation was confirmed by assessing the pedal withdraw reflex in the hind limbs, and the toe pinch and eyelid reflex^25^. The time between anaesthetic induction and image acquisition was similar, approximately 5–10 min, in all the experimental groups.

2-D M-mode echocardiography and tissue and pulsed wave Doppler imaging were performed using a MS 250 transducer (13–24 MHz) connected to a Vevo 2100 echocardiograph (VisualSonics, Canada). Animals were placed in a supine position on a heating pad to maintain the core body temperature between 37.5–37.7 °C, measured using a rectal probe. Body temperature was monitored during the whole procedure to prevent anaesthesia-induced hypothermia and to assess the anaesthesia depth. ECG recordings were performed to monitor the heart and breathing rate. Anaesthesia dosage was modified if needed to ensure similar heart and breathing rates between all animals. Heart rate, end-diastolic- and systolic diameters, as well as left ventricular anterior wall thickness and posterior wall thicknesses in diastole and systole, were acquired on the parasternal short-axis using M-mode imaging. Stroke volume, fractional shortening, ejection fraction, cardiac output, as well as end-diastolic and -systolic volume (Teichholz formula), were calculated based on M-mode images. Left ventricular filling was assessed by pulsed wave Doppler trans-mitral flow tracings (gate size 0.29 mm and Doppler angle − 25°), including E, A, isovolumic contraction time, aortic ejection time, isovolumic relaxation time, and mitral valve deceleration time, just above the tip of the mitral valve leaflets using an apical view. Non-flow time was the sum of isovolumic contraction, aortic ejection, and isovolumic relaxation time. Systolic peak wave, E′, and A′ were measured with tissue Doppler imaging (gate size 0.29 mm and Doppler angle 0°) at the lateral mitral annulus using an apical view. To assess diastolic function, E/A, E/E′, E′/A′, and E/E′/SV ratios were calculated. Myocardial performance index, an indicator for systolic and diastolic function, was calculated by dividing summed isovolumic contraction and relaxation time by aortic ejection time. In addition to presenting the non-indexed values, cardiac volumes were corrected for BSA to account for differences in body weight between the obese and lean ZSF1 rats. At least three stable cardiac cycles were averaged for all measurements.

After the procedure, ketamine/xylazine-anaesthetised animals were recovered by injecting 0.1 mg/kg atipamezole (Antisedan, Elanco Animal Health, Antwerpen, Belgium) intraperitoneally, while maintaining their body temperature between 37.5–37.7 °C to prevent anaesthesia-induced hypothermia.

### Statistical analysis

Results are presented as mean ± SD. Statistical analysis was performed using GraphPad software V7 (San Diego, CA, USA). Normal distribution of all continuous variables was tested according to the D′Agostino & Pearson omnibus normality test. Normal distributed pressure-volume loop data were analysed using a two-tailed unpaired Student t-test, while non-normally distributed data were analysed by a Mann-Whitney U test. Echocardiography parameters were analysed by regular two-way analysis of variance (ANOVA) with Šídák’s multiple comparison post hoc test.* P* values of < 0.05 were considered statistically significant.

## Supplementary information

Supplementary Information.

## Data Availability

All generated and analysed data during the study are included in the article and the supplementary information files. The raw data that support the findings of this study are available from the corresponding author upon reasonable request.
