# Supplementary material for: The effect of different anaesthetics on echocardiographic evaluation of diastolic dysfunction in a heart failure with preserved ejection fraction model
Source: Sci Rep. 2020 Sep 24;10:15701. doi: 10.1038/s41598-020-72924-5 (PMC7518268; doi:10.1038/s41598-020-72924-5)
Supplement: Supplementary file 1 — Supplementary Information. [file 41598_2020_72924_MOESM1_ESM.pdf]

The effect of different anaesthetics on echocardiographic evaluation of diastolic dysfunction in a heart failure with preserved ejection fraction model.

Ilona Cuijpers[a,b], Paolo Carai[a], Pedro Mendes-Ferreira[c,d], Steven J. Simmonds[a], Paul Mulder[e], Daniela Miranda-Silva[d], Daria De Giorgio[a,f], Peter Pokreisz[a], Stephane Heymans[a,b,g], and Elizabeth A.V. Jones[a,b*].

[a] Centre for Molecular and Vascular Biology, Department of Cardiovascular Sciences, KU Leuven, Herestraat 49, bus 911, 3000 Leuven, Belgium

[b] Department of Cardiology, Maastricht University, CARIM School for Cardiovascular Diseases, Universiteitssingel 50, 6229 ER Maastricht, Netherlands

[c] Department of Chronic Diseases, Metabolism & Aging, KU Leuven, Respiratory Division, Herestraat 49, bus 707, 3000 Leuven, Belgium

[d] Department of Physiology and Cardiothoracic Surgery, University of Porto, Cardiovascular R&D Unit, Faculty of Medicine, Alameda Prof. Hernâni Monteiro, 4200-319 Porto, Portugal

[e] Institut National de la Santé et de la Recherche Médicale (Inserm) UMR1096, Faculty of Medicine and Pharmacy, 22 Boulevard Gambetta, 76183 Rouen, France

f Department of Cardiovascular Medicine, Laboratory of Cardiopulmonary Pathophysiology, Istituto di Ricerche Farmacologiche "Mario Negri" IRCCS, Via Mario Negri 2, 201566, Milan, Italy

[g] ICIN-Netherlands Heart Institute, Holland Heart House, Moreelsepark 1, 3511 EP Utrecht, Netherlands

Corresponding author:

Prof. Elizabeth A V Jones, PhD

Centre for Molecular and Vascular Biology, Department of Cardiovascular Sciences, KU Leuven, Herestraat 49, bus 911, 3000 Leuven, Belgium

E-mail liz.jones@kuleuven.be

Supplementary Figures

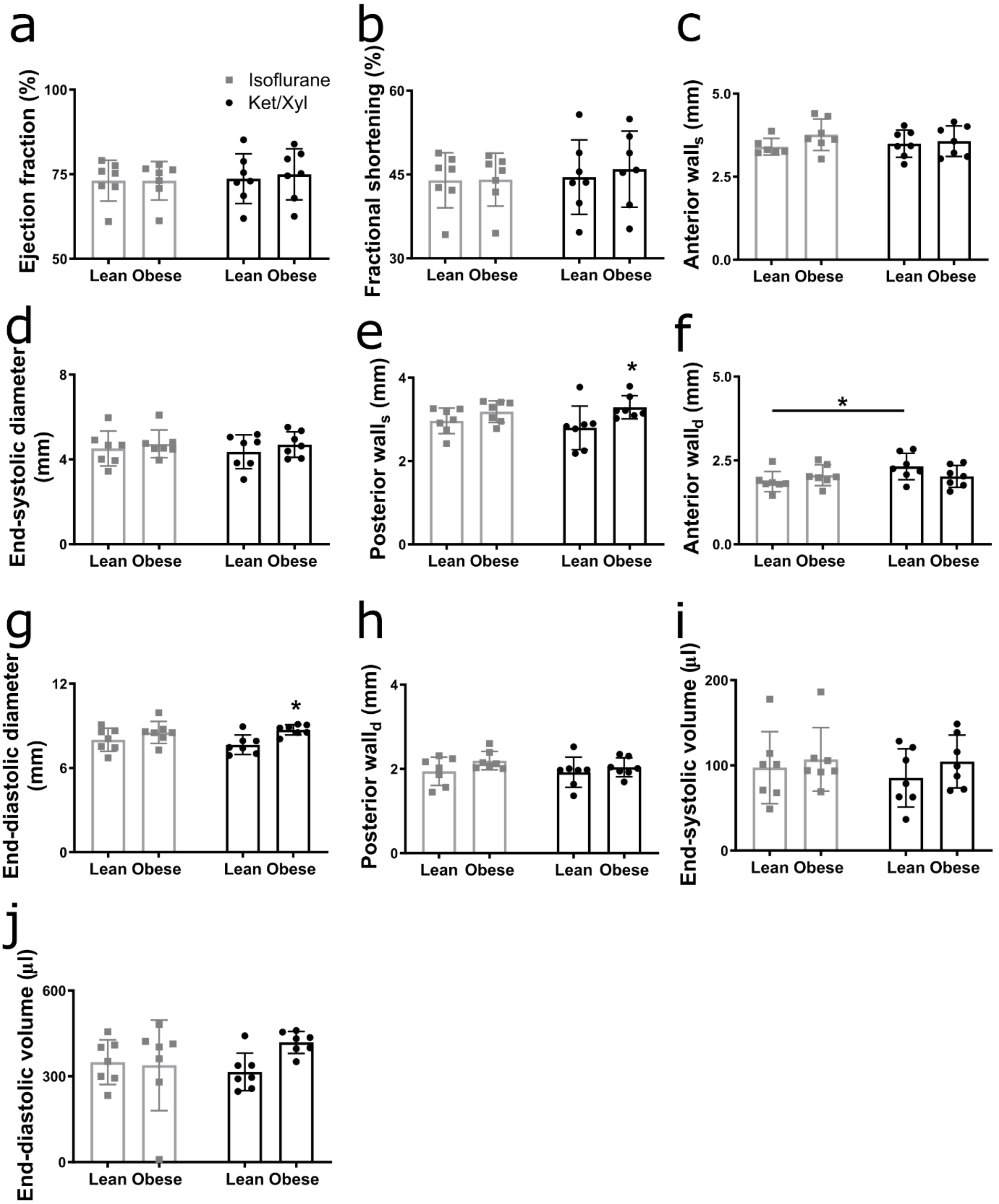

Supplementary Figure S1- The effects of ketamine/xylazine on systolic function and left ventricular dimension and volumes. Ejection fraction (**a**), fractional shortening (**b**), anterior wall thickness in systole (**c**), end-systolic diameter (**d**), posterior wall thickness in systole (**e**), anterior wall thickness in diastole (**f**), end-diastolic diameter (**g**), posterior wall thickness in diastole (**h**), and end-systolic and -diastolic volumes (**i**,**j**) in in 20-week-old isoflurane- or ketamine/xylazine-anaesthetised lean and obese ZSF1 rats (n=7 per group). Values are presented as mean ± SD. *P<0.05. $_d$, in diastole; Ket/Xyl, ketamine/xylazine; $_s$, in systole.

Supplementary Tables

Supplementary Table S1- Echocardiography parameters in isoflurane- or ketamine/xylazine-anaesthetised lean and obese ZSF1 rats

Parameter	Lean-Isoflurane (n=7)	Obese-Isoflurane (n=7)	Lean-Ket/Xyl (n=7)	Obese-Ket/Xyl (n=7)
**Heart rate (bpm)**	360 ± 20	310 ± 26***	243 ± 25###	200 ± 20**,###
**Stroke volume (µl)**	252 ± 43	287 ± 41	230 ± 42	314 ± 46**
**Stroke volume, $_i$ (µl/cm^2)**	0.51 ± 0.09	0.47 ± 0.07	0.50 ± 0.14	0.52 ± 0.08
**Cardiac output (ml/min)**	90 ± 12	88 ± 14	55 ± 7.3###	63 ± 12##
**Cardiac index (ml/min/cm^2)**	0.18 ± 0.02	0.14 ± 0.03*	0.12 ± 0.02###	0.11 ± 0.02##
**Anterior wall$_d$ (mm)**	1.9 ± 0.3	2.1 ± 0.3	2.3 ± 0.4#	2.0 ± 0.3
**Anterior wall$_s$ (mm)**	3.4 ± 0.3	3.8 ± 0.5	3.5 ± 0.4	3.6 ± 0.5
**End-diastolic diameter (mm)**	8.0 ± 0.8	8.5 ± 0.8	7.7 ± 0.7	8.7 ± 0.4*
**End-systolic diameter (mm)**	4.5 ± 0.8	4.7 ± 0.7	4.4 ± 0.8	4.7 ± 0.6
**Posterior wall$_d$ (mm)**	1.9 ± 0.3	2.2 ± 0.2	1.9 ± 0.4	2.0 ± 0.3
**Posterior wall$_s$ (mm)**	3.0 ± 0.3	3.2 ± 0.3	2.8 ± 0.5	3.3 ± 0.3*
**End-diastolic volume (µl)**	350 ± 78	391 ± 61	316 ± 66	419 ± 39
**End-diastolic volume, $_i$ (µl/cm^2)**	0.70 ± 0.16	0.64 ± 0.11	0.68 ± 0.20	0.70 ± 0.06
**End-systolic volume (µl)**	97 ± 42	107 ± 37	85 ± 34	105 ± 31
**End-systolic volume, $_i$ (µl/cm^2)**	0.20 ± 0.08	0.18 ± 0.06	0.18 ± 0.08	0.10 ± 0.05
**Ejection fraction (%)**	73 ± 6.0	73 ± 5.7	74 ± 7.4	75 ± 7.5
**Fractional shortening (%)**	44 ± 4.9	44 ± 4.8	45 ± 6.7	46 ± 6.8
**Aortic ejection time (ms)**	74 ± 7.5	84 ± 7.4	74 ± 9.9	103 ± 13***,##
**Isovolumic contraction time (ms)**	19 ± 3.0	19 ± 2.8	15 ± 3.3	18 ± 2.8
**Non-flow time (ms)**	125 ± 14	141 ± 11	127 ± 13	167 ± 14***,##
**Myocardial performance index (AU)**	0.69 ± 0.05	0.67 ± 0.05	0.68 ± 0.04	0.62 ± 0.04*
**Systolic peak wave (mm/s)**	45 ± 13	37 ± 4.0	40 ± 3.0	33 ± 9.8
**E'/A'**	1.7 ± 0.8	1.3 ± 0.4	2.3 ± 0.84	3.0 ± 0.53*,###

Values are presented as mean ± SD. A', late mitral annulus peak velocity; $_d$, in diastole; E', early diastolic mitral annulus peak velocity; $_i$, indexed for body surface area, calculated as 9.1 * body weight in grams$^{2/3}$; $_s$, in systole. *P<0.05, **<0.01, and ***<0.001 comparing ketamine/xylazine- or isoflurane-anaesthetised Lean versus Obese rats. #P<0.05, ##<0.01, and ###P<0.001 comparing isoflurane- versus ketamine/xylazine-anaesthetised in each group of rats (either Lean or Obese).

Supplementary table S2- Echocardiography parameters of isoflurane-anaesthetised lean and obese ZSF1 rats treated with or without ivabradine.

**Parameter**	**Lean Saline (n=7)**	**Obese Saline (n=8)**	**Lean +Ivabradine (n=7)**	**Obese +Ivabradine (n=8)**
**Heart rate (bpm)**	369 ± 47	317 ± 28*	302 ± 41$^{\#\#\#}$	258 ± 19$^{*,\#\#\#}$
**Stroke volume (µl)**	291 ± 18	328 ± 31	399 ± 62$^{\#}$	359 ± 31
**Stoke volume, $_i$ (µl/cm^2)**	0.53 ± 0.04	0.50 ± 0.09	0.61 ± 0.11	0.54 ± 0.10
**Cardiac output (ml/min)**	108 ± 16	104 ± 12	101 ± 23	93 ± 8.7$^{\#}$
**Cardiac index (ml/min/cm^2)**	0.19 ± 0.03	0.16 ± 0.03	0.18 ± 0.04	0.14 ± 0.03*
**Anterior wall$_d$ (mm)**	2.1 ± 0.2	2.1 ± 0.2	2.1 ± 0.3	2.1 ± 0.1
**Anterior wall$_s$ (mm)**	4.1 ± 0.3	3.9 ± 0.3	4.0 ± 0.3	3.8 ± 0.3
**End-diastolic diameter (mm)**	8.1 ± 0.1	8.8 ± 0.3**	8.5 ± 0.6	9.1 ± 0.3*
**End-systolic diameter (mm)**	3.7 ± 0.3	4.4 ± 0.2**	3.9 ± 0.5	4.7 ± 0.4***
**Posterior wall$_d$ (mm)**	2.1 ± 0.4	2.0 ± 0.2	2.0 ± 0.2	2.0 ± 0.2
**Posterior wall$_s$ (mm)**	3.4 ± 0.4	3.2 ± 0.2	3.5 ± 0.2	3.3 ± 0.2
**End-diastolic volume (µl)**	352 ± 12	421 ± 32**	398 ± 63	460 ± 36*
**End-diastolic volume, $_i$ (µl/cm^2)**	0.63 ± 0.03	0.64 ± 0.10	0.72 ± 0.12	0.69 ± 0.11
**End-systolic volume (µl)**	61 ± 11	93 ± 13**	66 ± 19	101 ± 19***
**End-systolic volume, $_i$ (µl/cm^2)**	0.11 ± 0.02	0.14 ± 0.02*	0.12 ± 0.03	0.15 ± 0.02
**Ejection fraction (%)**	83 ± 3.3	79 ± 2.3	84 ± 3.6	78 ± 3.5**
**Fractional shortening (%)**	54 ± 3.7	50 ± 2.3	55 ± 4.3	48 ± 4.0**
**Isovolumic contraction time (ms)**	17 ± 2.3	16 ± 2.4	17 ± 3.0	17 ± 3.4
**Aortic ejection time (ms)**	63 ± 6.0	82 ± 2.3***	66 ± 4.3	90 ± 3.5$^{***,\#\#\#}$
**Non-flow time (ms)**	106 ± 7.8	133 ± 3.7***	112 ± 5.2$^{\#}$	143 ± 3.8$^{***,\#\#\#}$
**Myocardial performance index**	0.70 ± 0.08	0.63 ± 0.05	0.70 ± 0.08	0.61 ± 0.06*
**E'/A'**	0.60 ± 0.17	0.86 ± 0.40	0.69 ± 0.14	1.2 ± 0.3**
**Systolic peak wave (mm/s)**	42 ± 12	38 ± 5.7	48 ± 15	41 ± 5.1

Values are presented as mean ± SD. A', late mitral annulus peak velocity; $_d$, in diastole; E', early diastolic mitral annulus peak velocity; $_i$, indexed for body surface area, calculated as 9.1 * body weight in grams$^{2/3}$; $_s$, in systole.*P<0.05, **<0.01, and ***<0.001 comparing either isoflurane-anaesthetised Lean and Obese treated with saline or isoflurane-anaesthetised Lean and Obese rats treated with ivabradine. $^{\#}$P<0.05, $^{\#\#\#}$<0.01, and $^{\#\#\#}$P<0.001 comparing ivabradine- versus non-ivabradine-treated isoflurane-anaesthetised in each group of rats (either Lean or Obese).

Supplementary Table S3- Comparison of Ket/Xyl and isoflurane on echocardiography parameters in rats

Condition<br>Strain	Age(wk) /weight(g)/<br>Gender (M/F)	Ket<br>(mg/kg)	Xyl<br>(mg/kg)	Isoflurane<br>(%)	Outcome<br>(Ket/Xyl vs Isoflurane)	Reference
**Healthy<br>Wistar**	Adult/275-325/M	0.1	0.75	1.5	HR ↓<br>CO ↓<br>SV =<br>EF =<br>EDD and ESD =<br>PW_d =<br>E ↓<br>A ↓<br>E/A =	[1]
**Healthy<br>Sprague Dawley**	Adult/330/M	30	5	1.5	HR ↓<br>CO =<br>SV =<br>EF =<br>EDD and ESD =<br>PW_d ↑<br>EDV and ESV =<br>S'=	[2]
**Healthy<br>Sprague Dawley**	10/ND/M	50	10	1.5	HR ↓<br>EDD =<br>ESD = (15/30'), ↓ (45')<br>EF =<br>EDV = (15/30'), ↓ (45')<br>ESV = (15/30'), ↓ (45')	[3]
**Healthy<br>Fischer 344**	9-12/199-290/M	37	7	1.5	CO ↓<br>SV =<br>EF =<br>EDD ↓<br>AW_d =<br>E =<br>A =<br>E/A =<br>DT =<br>IVRT ↑<br>EDV ↑<br>AET ↑	[4]
**Healthy<br>Wistar**	9/298-306/M	40	8	2.0	HR↓<br>CO ↓<br>EF =<br>EDD ↑<br>E =<br>A ↓<br>E/A ↑<br>DT =<br>E' ↓<br>E/E' ↓	[5]
**Chronic Aortic Regurgitation<br>Wistar**	Adult/275-325/M	0.1	0.75	1.5	HR ↓<br>CO ↓<br>SV =<br>EF =<br>EDD and ESD↓<br>PW_d =<br>E ↓<br>A =<br>E/A =	[1]

A, late mitral inflow peak velocity; AET, aortic ejection time; AW, anterior wall; CO, cardiac output; d, in diastole; DT, deceleration time; E, early mitral inflow peak velocity; E', early diastolic mitral annulus peak velocity; EDD, end-diastolic diameter; EDV, end-diastolic volume; EF, ejection fraction; ESD, end-systolic diameter; ESV, end-systolic volume; F, female; HR, heart rate; IVRT, isovolumic relaxation time; Ket, ketamine; LVID, left ventricular internal diameter; M, male; PW, posterior wall; s, in systole; S', systolic peak wave; SV, stroke volume; Xyl, xylazine. 'indicates the time after the anaesthesia induction.

References

1 Plante, E. *et al.* Impact of anesthesia on echocardiographic evaluation of systolic and diastolic function in rats. *J Am Soc Echocardiogr* **19**, 1520-1525, doi:10.1016/j.echo.2006.06.011 (2006).

2 Redfors, B., Shao, Y. & Omerovic, E. Influence of anesthetic agent, depth of anesthesia and body temperature on cardiovascular functional parameters in the rat. *Lab Anim* **48**, 6-14, doi:10.1177/0023677213502015 (2014).

3 Sano, Y. *et al.* Effects of various types of anesthesia on hemodynamics, cardiac function, and glucose and lipid metabolism in rats. *Am J Physiol Heart Circ Physiol* **311**, H1360-H1366, doi:10.1152/ajpheart.00181.2016 (2016).

4 Stein, A. B. *et al.* Effects of anesthesia on echocardiographic assessment of left ventricular structure and function in rats. *Basic Res Cardiol* **102**, 28-41, doi:10.1007/s00395-006-0627-y (2007).

5 Droogmans, S. *et al.* Impact of anesthesia on valvular function in normal rats during echocardiography. *Ultrasound Med Biol* **34**, 1564-1572, doi:10.1016/j.ultrasmedbio.2008.02.017 (2008).
